# Impact forces in backward falls: Subject-specific video-based rigid body simulation of backward falls

**DOI:** 10.1177/09544119231207653

**Published:** 2023-11-16

**Authors:** Fatemeh Khorami, Numaira Obaid, Tim Bhatnagar, Ahmed Ayoub, Steve N Robinovitch, Carolyn J Sparrey

**Affiliations:** 1 Mechatronic Systems Engineering, Simon Fraser University, Surrey, BC, Canada; 2 International Collaboration on Repair Discoveries (ICORD), Vancouver, BC, Canada; 3 Orthopaedics, University of British Columbia, Vancouver, BC, Canada; 4 Biomedical Physiology and Kinesiology, Simon Fraser University, Burnaby, BC, Canada

**Keywords:** Fall, rigid body dynamics, Madymo, older adults’ fall, joint stiffness

## Abstract

A critical missing component in the study of real-world falls is the ability to accurately determine impact forces resulting from the fall. Subject-specific rigid body dynamic (RBD) models calibrated to video captured falls can quantify impact forces and provide additional insights into injury risk factors. RBD models were developed based on five backward falls captured on surveillance video in long-term care facilities in British Columbia, Canada. Model joint stiffness and initial velocities were calibrated to match the kinematics of the fall and contact forces were calculated. The effect of joint stiffnesses (neck, lumbar spine, hip, and knee joint) on head contact forces were determined by modifying the calibrated stiffness values ±25%. Fall duration, fall trajectories, and maximum velocities showed a close match between fall events and simulations. The maximum value of pelvic velocity difference between Kinovea (an open-source software 2D digitization software) and Madymo multibody modeling was found to be 6% ± 21.58%. Our results demonstrate that neck and hip stiffness values have a non-significant yet large effect on head contact force (*t*(3) = 1, *p* = 0.387 and *t*(3) = 2, *p* = 0.138), while lower effects were observed for knee stiffness, and the effect of lumbar spine stiffness was negligible. The subject-specific fall simulations constructed from real world video captured falls allow for direct quantification of force outcomes of falls and may have applications in improving the assessment of fall-induced injury risks and injury prevention methods.

## Graphical abstract

**Graphical abstract:**
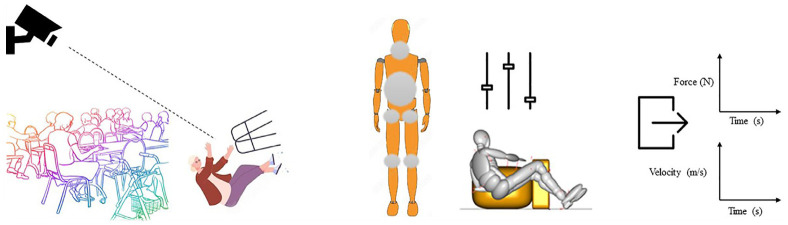

## Introduction

Falls are one of the most common causes of injuries in older adults, with approximately one in three individuals over the age of 65 falling at least once each year.^1,2^ Falls from a standing height account for 60% of traumatic brain injuries.^3^ An important limitation when investigating falls is the inability to directly quantify the impact forces experienced by an individual during a real fall event. Quantifying these forces is important because they are a key determinant of whether a fall results in injury.^4^

Fall studies have employed a variety of techniques to extract forces and velocities but are unable to directly measure forces during an injury. For example, falls may be mimicked in a controlled laboratory setting to measure the impact forces using force plates.^5^ However, for safety, the impact energies (and forces) are maintained below the energies involved in real-life falls and are rarely conducted in at-risk populations such as older adults.^6–8^ Planar videos of real-life falls of older adults in long-term-care facilities are becoming accessible for research and include injurious events.^9,10^ Video-extracted fall kinematics show good agreement with actual kinematics^11,12^ and observed injuries.^5^ However, only joint angles, heights, and velocity of the fall can be extracted^13,14^ not impact forces. New methods are needed to accurately quantify the forces associated with falls and injury events for older adults and assess injury prevention approaches.

Rigid body simulations are an effective tool to estimate the external and internal forces of a biomechanical system during a fall event.^15–18^ These models are used to reconstruct fall events for accident investigation,^18^ to conduct parametric studies to identify factors that influence fall biomechanics^19^ and to assess strategies for injury prevention.^20^ A major limitation of previous rigid body simulations of fall events is the use of parameter values that have only been validated for forces and velocities associated with car crashes.^21^ Furthermore, in most simulations, the kinematics are approximated,^22^ not directly calibrated to the actual injury event introducing additional inaccuracies. Default model parameters, such as joint stiffness values may not adequately represent older adult joint stiffness during a fall. Joint stiffness and initial joint velocities affect fall duration and impact intensity and are likely to affect impact force.^23–25^ In a study on backward falls, by altering leg joint stiffness, the falling period and impact velocity varied significantly.^26^ Initial joint velocities were also influential on falling dynamics impact mechanics.^25^ Therefore, joint velocities and individual joint stiffness are expected to be important for accurately predicting impact forces resulting from backward falls.

The main objectives of this study were to assess the accuracy of rigid body dynamics (RBD) models in capturing fall kinematics and to quantify the effect of assigned joint stiffness (neck, lumbar spine, hip, and knee) on head contact force. Additionally, the study aimed to develop a new method for extracting impact forces from real-world falls by reconstructing video footage of backward falls to quantify the injury kinematics and kinetics of older adult falls. Quantifying impact forces associated with real-world falls in older adults will provide additional insights into the causes of injury and opportunities to reduce injuries with fall protection and injury prevention strategies.

## Materials and methods

### Rigid body dynamic models

**Figure 1.**
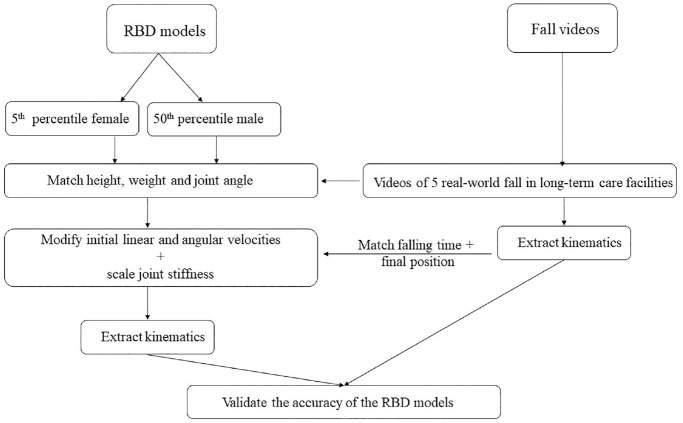
Diagram showcasing the process of the comparative study. This involved generating Rigid Body Dynamics (RBD) models via visual examination of videos, followed by a validation process where these models were cross-referenced against fall kinematics derived from a standard video kinematic analysis.

Rigid body dynamic simulations of each fall event were constructed (Madymo, TASS International, Livonia, MI). Validated dummy models were anthropometrically scaled from either the 50th percentile Hybrid III dummy model for males or the fifth percentile Hybrid III dummy model for females to match the height and weight of each participant. Objects involved in the fall, such as furniture or walls, were included in the simulation to replicate the fall conditions. Each fall simulation was constructed to match the last active posture before falling under the force of gravity. Videos of the actual falls were reviewed to identify the video frame where the participant showed their last voluntary motion (step, reach, etc). The corresponding video frame for both camera angles was selected. Simulations were constructed using view angles matching the video-captured camera angles. Still frames of the simulation geometry were overlayed on each of the video frame perspectives and the joint angles (wrist, elbow, shoulder, spine, hip, knee, and ankle) were modified to match the participant positioning. Figure 1 illustrates the step-by-step process followed in the study for constructing rigid body dynamic models and validating them using video kinematic analysis.

Initial linear and angular velocities were applied to the dummy center of gravity and major joints (hip, knee, ankle, shoulder, and elbow) involved in the fall. Initial velocities and joint stiffnesses were calibrated by overlaying the frame by frame progression of the falling participant and simulation. The initial velocity values of the lower arm (W1, W2), shoulder (W1, W2), hip (W1, W2, W3), lumber spine (W1, W2, W3), knee (W1), and overall dummy (W1, W2, W3, V1, V2, V3) were adjusted to match the motion of the recorded video. The velocities were fine-tuned by altering in increments of +100%, +50%, −50%, and −100% for different body parts, ensuring a comprehensive range of modifications. The overall dummy, smaller adjustments of +20%, +10%, −10%, and −20% were applied to the initial velocity parameter.^19^ Applying initial velocity conditions to the joints simulated the response of the individual during the fall. The neck, lumbar spine, hip, and knee joint stiffness were scaled from their default values in Madymo to visually calibrate the simulated fall with the video recording to better capture the effects of muscle activation on joint stiffness (Table 1).

**Table 1.** Modified scaling factors of joint stiffnesses for neck, lumbar spine, hip, and knee joints for five falls.

Fall	Neck	Lumbar spine	Hip	Knee
Fall #1	1.07	0.92	0.87	1.08
Fall #2	1.60	1.01	0.95	1.16
Fall #3	1.77	1.02	1.07	1.44
Fall #4	1.77	1.02	1.07	3.28
Fall #5	2.02	1.57	1.43	1.67

**Figure 2.**
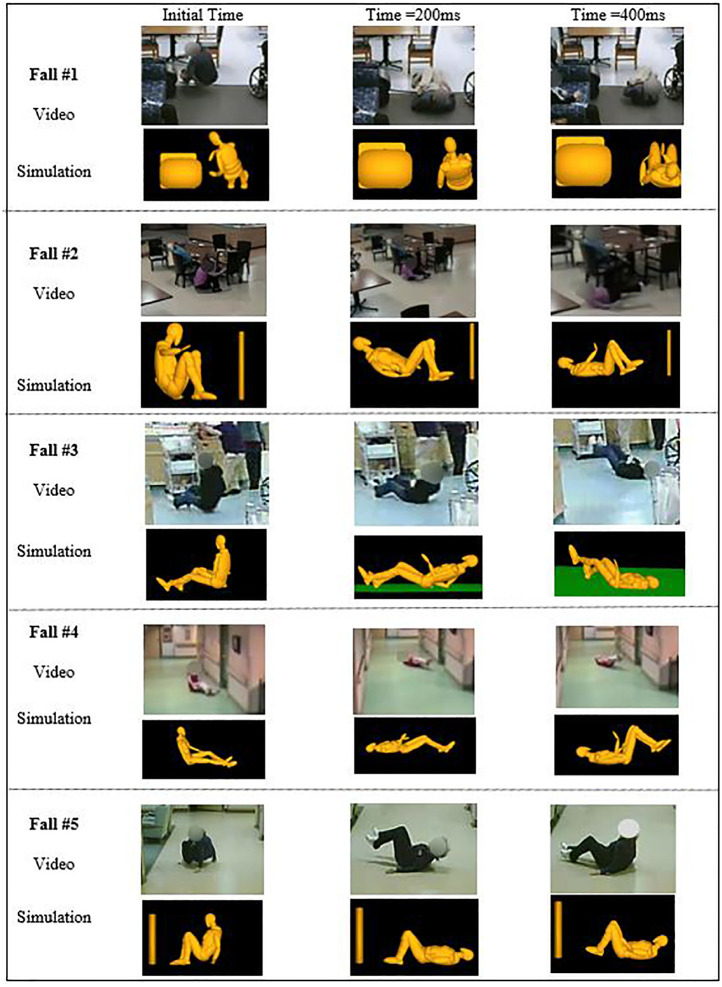
Comparing the simulation with final conditions against video in all five falls.

In our study, the modeling of soft tissue involved the utilization of default validated values for the material properties, as specified by Madymo, in the force-based contact characteristics for the ellipsoids.^27^ To enhance the realism of the model, specific modifications were incorporated, drawing upon empirical data. For the interaction of the pelvis with the floor, contact properties were derived from experimental data that accurately represented the overall contact stiffness of the pelvis during lower speed fall events.^28,29^ Similarly, for the head, the force-deflection characteristics were informed by data from head impacts.^30^ The flooring was assigned a stiffness similar to linoleum over concrete,^31^ with a frictional coefficient of 0.6.^32^ A hysteresis model was used to model the properties of the floor as viscoelastic with a hysteresis slope of 1e7, a stiffness of 4e6 N/m at loading, and 1.5e3 N/m at unloading. The frictional coefficients of external surfaces such as furniture were iteratively modified to match the motion of the person observed in the video. Pelvis and head ground contact forces and joint internal forces (forces that arise from the stabilizing muscles) of the fall at the neck, lumbar spine, and femur were calculated.^33^ Joint positions were extracted from the fall simulations at the initial position (last voluntary movement by the faller) until 400 ms after the initial position (Figure 2).

### Video kinematics analysis and simulation validation

The kinematics predicted by the simulation were compared to those extracted from the fall videos and the literature to validate the accuracy of the rigid body dynamic models. Two long-term care (LTC) facilities in British Columbia, Canada (New Vista Society Care Home, NV, and Delta View Life Enrichment Centre, DV) participated in this study by providing video footage of resident falls, for research purposes. For both facilities, each participant or proxy, at the time of admission, provided consent. Details for the camera networks, resolution, recording rates, and fall classification technique have been previously published.^13,34^ The research ethics boards of Simon Fraser University and Fraser Health Authority approved the study. The video data were screened to extract backward fall incidents with at least two camera angles. From the fall video library, five (n=5) videos capturing backward falling residents matched these inclusion criteria (Table 2). None of the falls captured in these videos resulted in reported injuries.

**Table 2.** The anthropometric data of the individuals and details of the fall event were captured for five real-world falls.

Fall #	Age	Height (cm)	Weight (kg)	Sex	BMI	Fall description
1	81	183	72.4	M	216	This individual experienced a backward fall from a standing height. While walking, the individual became imbalanced, and attempted to hold onto a nearby couch with their hand, but did not succeed in preventing the fall. During the fall, the legs of the individual were bent. Upon the initial impact, the hip contacted the floor while the back, neck, and head remained elevated. The back of the individual then contacted the floor.
2	88	152	45.4	F	19.7	The individual was squatting on the floor while holding a chair and got imbalanced, which resulted in the individual experiencing a backward fall. During the fall, the legs of the individual were bent, and the hip first contacted the floor, followed by the back.
3	68	172	46.5	F	15.7	The individual experienced a fall from a standing height. The individual became imbalanced. The pelvis impacted the floor and subsequently, the head impacted the floor. The individual’s hands were closed during the fall. Fall 3 and Fall 4 were the same individual falling on different days in different scenarios.
4	68	172	46.5	F	15.7	The individual experienced a backward fall from a standing height. The initial impact resulted in the hip and the left hand contacting the floor, followed by the lumbar spine and the head. The legs of the individual were straight during the impact.
5	80	147	49	F	22.7	The individual became imbalanced while walking and attempted tohold on to a nearby couch. The attempt seemed to work for a few seconds but eventually, the individual hip impacted the ground. The individual succeeded to prevent head impact by using her hands to arrest the fall.

The height and weight of each individual were used to scale the rigid body dynamic (RBD) dummy models (MADYMO, TASS Automotive, Helmond, Netherlands).

To determine the kinematics of real-world backward falls, we used five real-life fall videos from four individuals. Fall videos were cropped using the VLC media player (VideoLAN Client, the campus of the École Centrale Paris). Video data of the fall events were truncated to include data from 3 s before the first impact with the ground until 2 s after the person came to rest. Videos of falls were analyzed using Kinovea (Tracxn, France), an open-source video analysis tool, to manually digitize the pelvis positions during the fall trajectory. The pelvis was tracked at the joint centre,^35^ and to translate pixels into meters, the digitized coordinates underwent one-dimension video calibrations based on the height of the fallers.^11^

**Figure 3.**
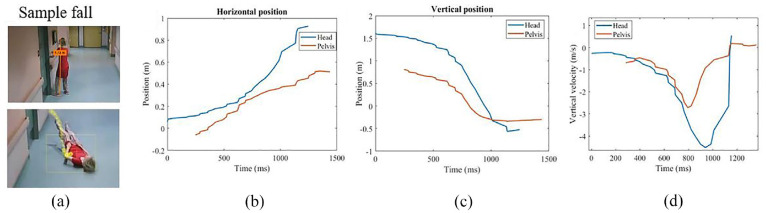
Digitization procedure: (a) calibration using the faller’s height. Examples of calibrated (b) horizontal and (c) vertical position versus time data in Kinovea. (d) Vertical velocity for head and pelvis in Kinovea.

The accuracy of determining the kinematics of falls using 2D analysis software, Kinovea, has been demonstrated in previous studies with video footage from consumer-grade devices.^11,36^ With this established validation, we have applied this software in our study to extract the pelvic velocity. The results from this extraction were then compared with the corresponding data from the Madymo simulations. To compare the actual falls with our simulations, we defined a specific window for comparison. The focal point of this window was the frame where the pelvis reached its minimum vertical distance from the ground, signifying the point of impact, as identified by an observer. This window, spanning 400 ms, enabled us to closely monitor the pelvic motion immediately before and after the impact. Within this window, we then determined the maximum pelvic velocity leading up to the impact. Figure 3 displays the Kinovea-derived kinematics, including the horizontal and vertical positions and velocities of both the head and pelvis. We then compared the maximum velocity within the defined window derived from both Kinovea and Madymo simulations using the equivalence testing (TOST) for statistical analysis.

### Sensitivity analysis of joint stiffness

For all falls, parametric studies were conducted to evaluate the effect of the neck, lumbar spine (LS), hip, and knee joint stiffnesses, which are known to increase with age. Stiffness values were altered from their calibrated values in MADYMO by ±25% to evaluate the effect of joint stiffness on the model’s ability to simulate the fall trajectory of older adults. The experimental design followed a three-level factorial design for each of the four joints selected resulting in 81 simulations for each participant. Within the simulation, we extracted the kinematic and kinetic data and subsequently compared the results with established injury criteria from the literature (Table 3).

**Table 3.** Injury threshold in the large male, mid-sized males, and small females.

Injury threshold	Large male	Mid-sized male	Small female
Neck Criteria			
Tension and Compression (N)	5440^a^	4500^a^	3370^a^
Femur Load (kN)	12.7^a^	10.0^a^	6.8^a^
	ll.537^b^		6.186^b^
Proximal Femur (N)			2826 ± 424 N^c^

a (57].

b (35).

c (33,34, 36–38, 51].

## Results

### Fall kinematics and model validation

The models accurately captured the timing and positioning of the hands, pelvis, and head during ground contact in all five falls (Table 4). Although we were not able to conclude that the means were equivalent (*p* = 0.406), the difference between Kinovea and Madymo maximum pelvis velocity values was low (6 ± 22%). This difference decreased in falls with a clear perpendicular view to the camera and no missing frames (3.4 ± 2.0% difference).

**Table 4.** Maximum pelvic velocity within a 400 ms time window centered around the moment of pelvis impact in Madymo and Kinovea (Madymo/max and Kinovea/max, respectively), and pelvic velocity at the time of impact in the Madymo simulations (Impact), shown for all five falls.

Fall	Pelvic		
	Madymo/max (m/s)	Impact (m/s)	Kinovea/max (m/s)
Fall # 1	1.17	0.80	1.11
Fall #2	0.92	0.24	0.74
Fall #3	0.86	0.20	1.29
Fall #4	3.03	2.52	2.99
Fall #5	1.27	0.60	1.76

### Contact and joint forces

**Figure 4.**
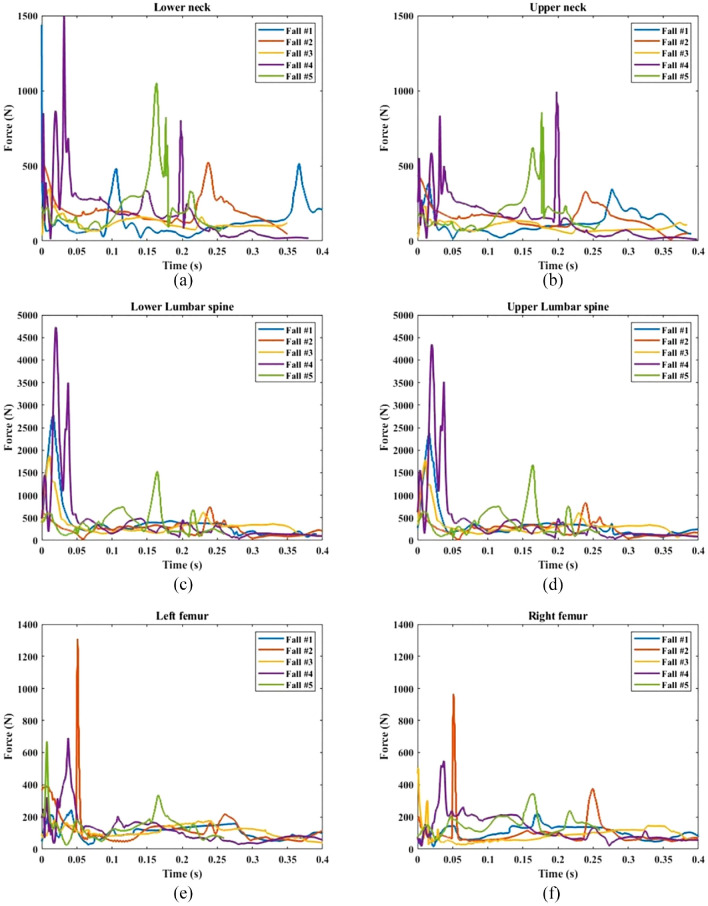
Force time history of: (a) lower neck, (b) upper neck, (c) lower lumbar spine, (d) upper lumbar spine, (e) left femur, and (f) right femur.

**Figure 5.**
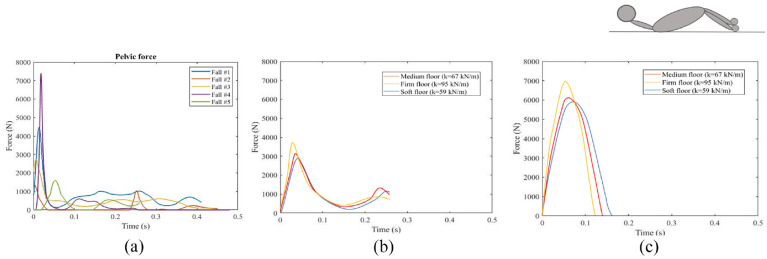
(a) Pelvic force in five falls in simulations. Hip impact force during a backward fall with different floor types using a (b) mathematical model, and (c) experimental setup on a force plate.^7,8^

Force time histories were extracted in Madymo (Figures 4 and 5). Internal neck, lumbar spine, and femur forces during the trajectory of the fall were reported as well as pelvis and head contact forces. Neck, lumbar-spine, and pelvic peak forces ranged from 775 *N* ± 437 N, 2253 *N*±1275N, and 3510 *N* ± 2215 N respectively. The femur force recorded in fall #2 was 1310 N, which represents an increase of 151% compared to the average femur force observed in the other falls (522 *N* ± 186 N). Maximum impact force was significantly affected by the fall height. As the fall height increased, the impact force likewise increased. Fall #4 showed higher peak forces in most body parts compared to other falls. A comparison between fall #4 and fall #2 indicated that straight knees resulted in significantly higher neck, lumbar spine, and pelvic forces compared to bent knees (187%, 561%, and 423% respectively, *p* < 0.001); however internal femur forces were significantly lower in fall #4 than fall #2 (*p* < 0.001). A similar effect was seen in a comparison of fall #4 and fall #5 where the faller arrested the fall with their hand. Hand contact significantly reduced forces in the neck, lumbar spine, and pelvis by 364%, 43%, and 210% respectively (*p* < 0.001). Body rotation in fall #1 significantly reduced pelvis force by 84% compared with fall #4 (*p* < 0.001). A trend was seen in the neck and lumbar spine forces reduction in fall #1 compared to fall #4 by 65%, and 159% respectively (*p* = 0.69 and *p* = 0.09 respectively).

### Parametric study

**Figure 6.**
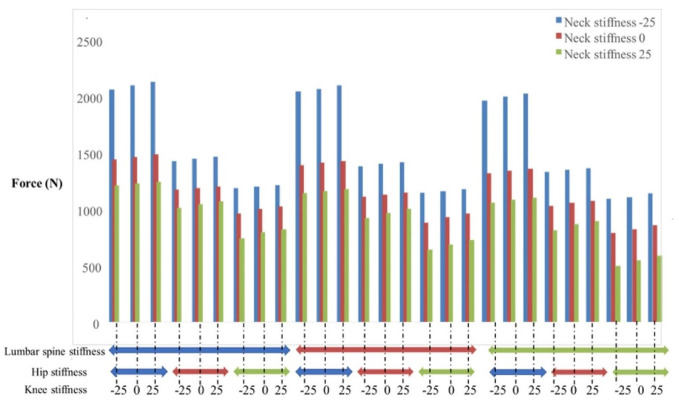
The effect of the neck, lumbar spine, hip, and knee stiffnesses on the head contact force in a sample fall. The stiffness effects were evaluated by a factorial design with values of −25%, 0%, and +25% of calibrated values in Madymo (total of 27 points) for each stiffness. *Blue, red and green colors indicate − 25%, 0%, and +25% stiffness scale respectively compared to the calibrated value.

Neck and hip stiffness were the two factors with the greatest effect on head contact force. The head contact force decreased as the stiffness of these joints increased (from 2390–945 N). Knee stiffness had a negligible effect on head contact forces (<2%). While lumbar spine stiffness increases resulted in small (<4%) decreases in the head contact force. The impact of hip and neck stiffness on head impact force was 150% higher than the impact of knee stiffness (Figure 6). In some simulations, the contact between the floor and the head was completely inhibited as a result of the combined increases in neck and hip stiffness.

## Discussion

Falls continue to be a significant source of injury for older adults despite decades of research and numerous injury prevention strategies. Video captured real-world falls have provided important insights into the specific circumstances of falls^13^ and can be used to extract fall kinematics.^11^ However, to understand injury risk it is important to be able to extract impact forces in addition to velocities. This study presents an approach to develop matched rigid body dynamic simulations to video captured real world falls to extract both kinematics and impact forces. By calibrating limb movements, joint stiffness, and body anthropometry to the video captured falls, the simulated models overcome the primary limitation of RBD models, which is the passive nature of the dummy models. Contact forces provide additional insights into the likelihood and severity of injuries using established injury criteria and thresholds.^37–44^ This is significant as experimental studies are limited to non-injurious forces and video analysis is limited to kinematic data and does not provide force outcomes. Subject-specific rigid body simulations of real fall events further provide the opportunity to conduct parametric studies to evaluate the effect of independent parameters on the force outcomes highlighting opportunities for quantitative assessment of injury prevention strategies.

Rigid body dynamic models provide the opportunity to systematically explore and quantify fall dynamics without risk to human subjects but themselves are limited by the accuracy of the model assumptions. Using Madymo as the modeling software, with its library of validated human models and the ability to anthropomorphically scale the human models helped to ensure the biofidelity of the simulations.^21^

In our study, we opted for the MADYMO Hybrid III pedestrian dummy due to its unique ability to emulate human biomechanics, particularly in the context of a standing posture necessary for the simulation of backward falls.^45^ This characteristic sets it apart from other crash test dummies such as frontal, side, and rear impact dummies, which lack this capacity. The pedestrian dummy has been designed and validated specifically for ground contact injury predictions and vertical impacts, directly aligning with the focus of our research.^46,47^

While previous models have been limited by the passive response of the dummy models,^15,17,18^ in this study we overcame that limitation by inputting initial movements to each joint and adapting joint stiffness to match video captured fall kinematics. Default contact characteristics of the models have been shown to overestimate impact forces in some cases;^48^ however, we found that implementing more realistic movements significantly reduced impact forces. Further work is needed to continue to improve the joint and contact characteristics of these models to better mimic lower intensity impacts as the majority of the properties in these RBD human models have only been validated for car crashes.^49^ The current study was limited to analyzing backward falls. It is not clear how the effects of joint stiffness might affect forward or lateral falls. The study of falls based on video footage of real-life falls has technical limitations including video quality and resolution, available camera angles, obstacles limiting the view, and the surrounding environment, which make precise fall analysis challenging.^13^ However, by coupling video analysis with simulation of these real-life falls, further analysis of factors affecting falls (i.e. joint stiffness, body posture, and active movement) are possible.

Although rigid body dynamic simulations have been used to explore a range of fall scenarios and the effects of joint positioning on fall kinematics, there have been limited opportunities for direct validation of modeling live human falls. Unlike Doorly and Gilchrist, who used default models and relied on eyewitness reports, often prone to inaccuracies, for setting initial conditions, we based our initial conditions on actual observed fall data.^15^ This direct calibration, and the careful consideration of individual differences in joint stiffness and initial joint velocities,^23–25^ allow for a more accurate representation of real-life fall dynamics. Comparatively, Schulz et al. estimated the severity of a fall from a bed by comparing simulated results with physical test data. They used an anthropomorphic test device (ATD) named Hybrid III and adjusted the model to replicate the impact event.^16^ However, their simulation focused on head impacts and required manual iteration to match the accelerometer data.^16^ Our study improves upon this by considering the whole body’s impact forces while validating our data. Adamec et al. used an iterative process, adjusting simulation parameters to align with real-world evidence. While practical, this approach can potentially introduce inaccuracies due to uncertainties in witness statements and forensic evidence.^17^ In Erickson et al. study, the model was calibrated to the 50th percentile Hybrid III dummy model, and it relied on optical tracking for setting initial conditions.^18^ However, this approach overlooks individual-specific characteristics, such as variations in joint stiffness and initial joint velocities.

In general, RBD models have not been developed or validated for low-energy fall events so care must be exercised in using this approach. Previous work from our group demonstrated the importance of including individual joint velocities in simulations.^25^ Small differences in model joint movements resulted in changes in head contact as well as up to a 40% difference in pelvic impact forces. Direct comparisons between fall velocities and predicted velocities in the current study demonstrated the capacity of the RBD approach for replicating fall kinematics. By prescribing center of gravity and individual limb movements and calibrating joint stiffness parameters to match fall kinematics with videos of real life falls, we were able to mimic an active fall response and directly validate each RBD model.

Previous studies demonstrated that for a variety of camera angles (two perpendicular video angles vs 60 and 30 degrees) and 1D video calibrations based on the fallers’ height, Kinovea resulted in acceptable accuracy for determining fall kinematics.^11^ For example, pelvic velocity showed the lowest errors in angular position and velocity, with an absolute difference of 0.12 ± 0.03 m/s in pelvic peak velocity between the 3D high-speed motion analysis camera and Kinovea during backward falls.^11^ However, errors became more significant when there were obstructions, missing frames or the fall was out of plane relative to the camera.^11^ Comparing extracted values from Kinovea and Madymo in this study showed no significant difference (*p* = 0.825) though we were also unable to establish equivalence (*p* = 0.406) due to variability in the simulations. The best match between the two methods was when we had a clear view throughout the fall trajectory, with a perpendicular view to the camera (3.42 ± 1.99% difference). The accuracy of kinematic data extracted from videos is affected by the precision of camera angles and resolution, obstructions, and clothing. Matching RBD simulations with video captured falls provides an opportunity to more accurately capture both fall kinematics and impact forces.

The quantitative observations of impact forces in our RBD simulations align well with general observations made in fall injury mechanics studies. For example, while similar head and pelvic velocities were observed in fall #4 and fall #5, arresting the fall with hand in fall #5 led to significantly lower neck forces which could prevent spine and head injuries. In a laboratory study on fall arrest strategies, hand/wrist contact was a common strategy to limit upper body and head contact with the ground.^50^ However, impact forces could not be extracted from these falls, and therefore direct quantitative force comparisons were not possible. A study of backward falls in stage combat experts found peak pelvic impact forces ranged from 1.9 to 3.8 times body weight (equivalent to 846– 2699 N for our subjects) in falls onto crash mats that did not result in injury.^51^ Three of the five simulations in this study showed pelvic impact forces within this range. However, fall #1 and fall #4 showed pelvic forces closer to 4400–7300 N (6 BW and 16 BW respectively). These higher forces align with backwards falls simulated for snowboarders that found ground impact forces ranging from 5060 to 7920 N.^52^ However, despite these high pelvic contact forces, femur forces in the same simulations were much lower. This may reflect the effect of different strategies employed during a fall to protect the body. For example, in fall #4, contact was primarily through the buttocks not directly on the hip. Therefore much of the impact may have been attenuated by the soft tissue and spine not translated to the femur. These observations reinforce the value of fall simulations in addition to kinematics or even contact forces. Specific fall strategies, body orientation, and body composition affect the translation of forces through the body and the resulting injury risk.

Relating forces to injury risk is an essential element of most fall biomechanics studies. However, for studies with human subjects, the translation of external forces through the body to specific structures (femur, spine, wrist, etc) can only be approximated but not directly calculated. Several groups have employed a range of computational approaches in trying to determine internal tissue loading corresponding to fall events including finite element models,^53,54^ spring-mass-damper lumped force models,^55^ and rigid body dynamic simulations.^18,30,56^ Each approach provides insights into tissue loading and injury risk; however, we are becoming increasingly aware of the need for these models to capture individual variability instead of a single generic computational model to accurately capture tissue mechanics. In this study, we found that default joint stiffness values for the human models needed to be scaled from 0.87 to 3.28 times the original values in order to match each fall’s kinematics. These joint stiffness values play an important role in load transmission through the body and resulting tissue loading.^26^ In addition, prescribing joint movements to simulate reaction to a fall was shown in our study to affect resulting impact forces. This agrees with simulations of falls from a height, which found initial joint velocities and joint reactions affected both the locations of body contact with the ground as well as resulting impact forces.^57^ While the calculated impact forces exceeded all injury thresholds and corresponded to catastrophic and fatal injuries, the authors concluded that the forces may have been excessive due to definitions of head contact properties. In our study, none of the five reconstructed falls resulted in reported injuries. This aligns with reported injury criteria for the neck, spine and femur (Table 4). Peak force values in the neck reached ∼1500 N, well below the injury threshold of 3370 N reported for small females.^38–42^ Further, femur forces for all reconstructed falls had a maximum value of 1300N, less than half of the proximal femur failure load reported for small females (2826 N).^37,41^ Although straight backward falls, such as fall #4 are often perceived to be more severe and have greater injury risk, our study found similar results to prior work on fall posture, that hip loading was greater when the torso was upright at impact than extended (fall #2 and fall #5 in this study).^35^ However, care must be taken in linking simulation forces to injury as previous studies reconstructing falls in Madymo found excessive forces in the neck and head which are attributed to overly stiff head contact properties in the dummy models.^15,57^ In the current study, modifications were made to the joint stiffness and velocity of baseline models which resulted in contact forces that better aligned with published results. However, additional work is still needed to better define soft tissue contact forces for these simulations which may result in even lower contact forces.

Our study, despite its significant insights, is not without limitations. One of these is the potential for error introduced through the interactive manipulation of simulation input data using Kinovea and during the tuning of dummy parameters for the rigid body dynamic simulations. This process, while essential to matching real-world fall kinematics, inherently introduces subjectivity and the potential for inconsistencies. Moreover, the challenges posed by the quality, resolution, and perspective of video footage and the limitations of the dummy models in replicating the nuanced properties of human soft tissue further add to the complexity. These limitations, although managed carefully in our study, point to areas for future refinement to enhance the robustness and accuracy of such research methodologies.

With increasing advances in computational models and increasing computational resources rigid body dynamic models provide an additional means to assess the efficacy of injury prevention strategies. Importantly, current testing paradigms are limited to isolated benchtop testing^58,59^ or generic modeling approaches that only use “average” statures.^60,61^ Soft tissues, exhibiting viscoelastic properties, absorb a greater proportion of force (1 mm of soft tissue can absorb as much as 70 N of force) during lower-speed incidents like falls, a scenario that may not be entirely accurately modeled with tools designed for higher-speed impacts like car crashes.^62^ Cadaveric specimens, which are limited to what is available and may not adequately capture the diversity of the living population, do not provide a link between fall mechanics and external loading experienced during fall related injuries making it difficult to accurately assess injury prevention methods. Further, the specific loading directions and intensities during fall events are variable and likely to differ from the prescribed loading of standardized tests; however, due to the limited ability to directly measure forces during injury events the specific forces contributing to fall related injuries are mostly still approximated from in vitro experiments. Reconstructing injury events using this coupled method of video capture and individualized rigid body dynamic analysis may provide new insight into the specific forces leading to injury.

## Conclusions

Coupling rigid body dynamic simulations with video captured real world falls provides the opportunity to extract external contact forces and well as internal body loading that may provide additional insights into injury risk and injury prevention. In order to match real world fall kinematics scaled human models were developed to match each individual’s height and weight. In addition, calibrating joint stiffness and velocity to the video captured fall were necessary to accurately capture the fall kinematics and resulting impact forces. As part of the sensitivity analysis for each simulated fall, we found that the risk of head impact was reduced with increasing neck and hip stiffness. We have shown that rigid body dynamic simulations of backward falls can accurately capture both fall kinematics as well as predict contact forces. This modeling approach provides the opportunity to quantitatively test injury prevention strategies such as hip protectors in a range of fall scenarios, on a range of body statures, and at impact forces likely to cause injury. This approach can complement standardized benchtop testing to better capture the real world efficacy of these injury prevention methods.
